# Severe atopic eczema and recurrent infections:diagnosis of the Job syndrome according to Guimbacher criteria

**DOI:** 10.1186/1939-4551-8-S1-A285

**Published:** 2015-04-08

**Authors:** Luiz Carlos Bandoli Gomes

**Affiliations:** 1 HU-Ufjf, Brazil

## Introduction

The syndrome of hyper-IgE syndrome or Job syndrome or Burckley syndrome, is a rare primary immunodeficiency characterized by defective phagocytic manifesting recurrent infections, mainly Staphylococcal associated with eczema severe atopic, musculoskeletal disorders, pathological fractures, scoliosis and levels high IgE (> 2.000UI / ml).

Among the immunological characteristics, we can find defects in neutrophil chemotaxis, decreased production of interferon gamma, eosinophilia mutation and STAT 3.Most cases are sporadic, but there are cases with patterns of autosomal dominant or recessive inheritance. The diagnosis is clinical using the criteria of Grimbacher.The treatment is the same of the bacterial and fungal infections. Some consensus oriented prophylaxis of infections with antibiotics.

## Objective

Report the case of a patient with severa atopic eczema and criteria for the syndrome hyper IgE Grimbacher seconds.

## Case description

IFS 2 years and 9 months, with a history of atopic eczema since 3 months of age and history of intrauterine growth retarded neonatal sepsis associated with severe skin lesions, three hospitalizations for pneumonia and hospitalization due infected with abscesses dermatitis. Physical examination showed coarse facies, prominent forehead, broad nasal bridge, hyperextensible joints of knees and severe atopic eczema refractory to treatments. In evaluating Additional research showed anti-HIV antibody negative, eosinophilia persistent (4080 cells / mm 3) and IgE> 2.000UI / ml.

## Conclusion

Given the difficulty for the molecular diagnosis of the syndrome hyper-IgE, we report a probable case of the disease evaluated according to the criteria of Grimbacher be used in our service. Reiterates the importance of investigating immunodeficiency in children with severe atopic eczema associated with recurrent infections.

## Consent

Written informed consent was obtained from the patient for publication of this abstract and any accompanying images. A copy of the written consent is available for review by the Editor of this journal.
